# Northampton General Hospital

**Published:** 1905-08-19

**Authors:** 


### HOSPITAL ADMINISTRATION.

### CONSTRUCTION AND ECONOMICS.

### NORTHAMPTON GENERAL HOSPITAL.

Quarterly Meeting of Governors.

The quarterly meeting of the Governors of the Northampton General Hospital took place at the institution on the 5th inst. Mr. W. Whitton, one of the vice-presidents, was voted to the chair.

Small Cost of the New Hospital Buildings.

It appears from the report submitted by the Building Committee that the total receipts on building account amount to £29,120, and the total expenditure, including contractor, architects' fees, all other expenses, and the cost of the furniture (£2,000) was £34,308. The amount due to the bank was £5,260, towards which donations amounting to £2,600 were promised. The new buildings contain 164 beds, so that the cost per bed, including furniture and all expenditure, is only £209.

It had been suggested to the Board that the lifts in the new buildings should be electrified, and that subways should be constructed to connect the administration block with the new wings. Owing to the present financial position of the hospital, the further consideration of constructing subways and electrifying the lifts has been deferred by the Board for 12 months.

Sir Henry Burdett's Report.

As the result of his final inspection of the new buildings and of the reconstructed Administration Block, Sir Henry Burdett had sent in a report, extracts from which were as follows:—"Speaking generally, I consider the present condition of the whole of the buildings proves the excellence of the work done by Mr. Henry Martin, builder. With the exception of a crack in the ceiling here and there, and certain defects which I noticed in the woodwork in the corridor of communication, and in one place in the terrazzo pavement, the work has stood the test of one year's occupancy remarkably well. The reconstruction of the administration block, taken as a whole, is very satisfactory. It provides ample and good accommodation for the whole of the resident and nursing staff. I am strongly of opinion that it is highly desirable that the electrical and light department should be placed in the hands of a member of the medical profession who has studied this branch, for in this way the amount of real good which the new hospital can accomplish may surprise everybody within a very short time. I was glad to find that the patients in the paying wards testify very strongly to the kindness and attention of the nurses and the staff. I cannot conclude this report without congratulating the committee and governors upon the possession of one of the best, most modern, and up-to-date county hospitals, which has been erected at a cost so relatively small as to be remarkable in these days of extravagance in buildings, and which will always possess the merit of having been completed at a cost within the original estimate. Facts like these testify to the care, forethought, and supervision which has been exercised by the Building and other Committees, and to the efficiency of the architects and builder, all of whom are entitled to the generous acknowledgments of the governors, and of the inhabitants of the town and county of Northampton in particular."

A New Dietary.

The whole question of the hospital dietaries had been carefully considered by a special committee of medical and other members of the Board, and tables had been arranged, not only for the patients, but for the nursing and domestic staff as well. An endeavour had been made to introduce more variety, and for these purposes daily bills of fare had been drawn up. These new arrangements will involve additional expenditure, but they are considered by the Board to be most desirable.

Mr. H. Manfield moved the resolution regarding the commutation of the late matron's (Miss Pell's) annuity, and, seconded by Mr. Adnitt, it was carried.

A Resolution of Thanks.

Mr. F. G. Adnitt moved the following resolution:—"That the cordial and sincere thanks of the Court of Governors be given to Sir Henry Burdett, K.C.B., and to Dr. Richard Greene for their great help and liberality in the building of the new wings and the reconstruction of the administrative block of the Northampton General Hospital. To Sir Henry Burdett for his original suggestion, for his monetary assistance, and for his zealous energy in inducing others to imitate his generosity and enthusiasm, without which the work could not have been undertaken. To Dr. Greene for his generosity, not only in financial matters, but in affording the hospital the full benefit of his ripe experience, his resourcefulness in architectural designing, and his mastery of detail in hospital requirements and equipment, without which the new buildings and reconstruction of the old could not have been brought to such a pre-eminently successful completion except at a considerably increased expenditure. That the heartiest thanks of the court be given to Mr. F. W. Dorman, A.R.I.B.A., of Northampton, for his professional work in conjunction with Dr. Greene in the rebuilding of the Northampton General Hospital, the Court desiring to place on record their feeling that better or more economical plans for the requirements of the hospital could not have been produced, and their high sense of the courteous and generous manner in which Mr. Dorman always met the hospital authorities, and the fair and able way in which all matters concerning contractors and others were successfully negotiated." Mr. Adnitt said, as chairman of the building committee, he wished also to bear testimony to the excellent work of the Contractor (Mr. Henry Martin), and the courteous way in which Mr. Martin and Mr. Smith, who superintended the work, received the suggestions of the building committee. They had now a thoroughly up-to-date hospital, with large, airy, and well-lighted wards, and everything required for carrying on the work of such an Institution, and he believed the medical and nursing staffs were thoroughly well satisfied. The quarters for the nurses especially were a great improvement. Mr. Adnitt then thanked his colleagues on the building committee for their assistance during the rebuilding, and especially mentioned the Vice-Chairman, Mr. John Cooper, J.P., of Delapre, who devoted a great deal of time and trouble to the work. In concluding, Mr. Adnitt also spoke in the highest terms of the way in which Mr. Risbee, their secretary, had carried out his duties. The resolution was seconded by the Chairman, who endorsed the remarks made by Mr. Adnitt, and was carried unanimously.

More Help Wanted.

Mr. Adnitt expressed the hope that the general public would rally to the support of the hospital. They would want, he thought, about £1,200 more each year, and he hoped, after spending £30,000 on what was really a cheap hospital, costing only about £200 per bed against from £400 to £500 in some towns, that the public would see that they did not lack funds for maintenance. (Applause.)

